# Toll-Like Receptor Signaling and SIGIRR in Renal Fibrosis upon Unilateral Ureteral Obstruction

**DOI:** 10.1371/journal.pone.0019204

**Published:** 2011-04-22

**Authors:** Veronika Skuginna, Maciej Lech, Ramanjaneyulu Allam, Mi Ryu, Sebastian Clauss, Heni Eka Susanti, Christoph Römmele, Cecilia Garlanda, Alberto Mantovani, Hans-Joachim Anders

**Affiliations:** 1 Department of Nephrology, Medizinische Poliklinik, University of Munich, Munich, Germany; 2 Medizinische Klinik und Poliklinik I Grosshadern, University of Munich, Munich, Germany; 3 Istituto Clinico Humanitas and Fondazione Humanitas per la Ricerca, Rozzano, Italy; Institut National de la Santé et de la Recherche Médicale, France

## Abstract

Innate immune activation via IL-1R or Toll-like receptors (TLR) contibutes to acute kidney injury but its role in tissue remodeling during chronic kidney disease is unclear. SIGIRR is an inhibitor of TLR-induced cytokine and chemokine expression in intrarenal immune cells, therefore, we hypothesized that Sigirr-deficiency would aggravate postobstructive renal fibrosis. The expression of TLRs as well as endogenous TLR agonists increased within six days after UUO in obstructed compared to unobstructed kidneys while SIGIRR itself was downregulated by day 10. However, lack of SIGIRR did not affect the intrarenal mRNA expression of proinflammatory and profibrotic mediators as well as the numbers of intrarenal macrophages and T cells or morphometric markers of tubular atrophy and interstitial fibrosis. Because SIGIRR is known to block TLR/IL-1R signaling at the level of the intracellular adaptor molecule MyD88 UUO experiments were also performed in mice deficient for either MyD88, TLR2 or TLR9. After UUO there was no significant change of tubular interstitial damage and interstitial fibrosis in neither of these mice compared to wildtype counterparts. Additional in-vitro studies with CD90+ renal fibroblasts revealed that TLR agonists induce the expression of IL-6 and MCP-1/CCL2 but not of TGF-β, collagen-1α or smooth muscle actin. Together, postobstructive renal interstitial fibrosis and tubular atrophy develop independent of SIGIRR, TLR2, TLR9, and MyD88. These data argue against a significant role of these molecules in renal fibrosis.

## Introduction

Chronic kidney disease is usually associated with diffuse remodeling of the tubulointerstitial compartment. Tubular atrophy and interstitial fibrosis represent a common terminal pathway of various types of kidney diseases involving numerous pathomechanisms and effector elements [1] such as loss of peritubular vasculature and ischemia [2], epithelial-mesenchymal transition [3], recruitment of circulating fibrogenic progenitor cells [4], the release of growth factors and proapoptotic molecules [5], and interstitial inflammation driven by chemokine-mediated leukocyte recruitment [6]. The factors that orchestrate the onset, resolution or progression of renal fibrosis remain unclear.

Generally, tissue remodeling is the result of a danger response [7]. All cell types can recognize toxic, metabolic, infectious, mechanic or osmotic types of danger and elicit primary signals that trigger defense mechanisms and that alert other cells. For example, pathogens trigger the expression of proinflammatory cytokines and chemokines via the activation of Toll-like receptors (TLR) by pathogen-associated molecular patterns (PAMP) like LPS, lipopeptides or bacterial DNA [8]. It has now become clear that the TLR-dependent innate immune response is essential in mediating host defense to infection and to infection-related inflammation and tissue remodeling [7], [8]. Interestingly, TLRs can trigger inflammation and tissue remodeling also in the absence of pathogens or PAMPs [9]. Dying cells were found to elicit identical immune activation by the release of endogenous ‘danger-associated molecular patterns’ (DAMP) that have immunostimulatory effects and act as endogenous immune adjuvants [10]. As such TLRs seem to functionally act as universal danger recognition receptors that alert the immune system to tissue damage independent of the type of injury [10]. This universal mechanism also applies to kidney diseases [11]. For example, postischemic renal inflammation and tubular damage are significantly reduced in mice deficient for TLR2, TLR4 or MyD88, the main adaptor molecule for these TLRs [12], [13], [14], [15], [16]. Data from other acute kidney injury models further support the role of TLRs in renal danger signaling [17], [18]. Does this concept also apply to tissue remodeling in chronic kidney disease? Only few studies have yet addressed this issue experimentally. Wang, *et al.* reported that tubulointerstitial disease was reduced in wildtype kidneys transplanted into TLR2-, TLR4-, TLR2/4-, MyD88-, and TRIF-deficient recipients [19]. These data document a role for TLRs in alloimmunity against a renal graft but not necessarily about the role of TLR signaling inside the donor kidney. The unilateral ureteral obstruction (UUO) model is often used in the context of renal fibrosis [20], [21] and data from TLR2– or TLR4-deficient mice propose that TLR signaling contributes to postobstructive renal inflammation, tubular atrophy, and interstitial fibrosis [22], [23].

Negative regulators of TLR signaling limit innate immune activation and thereby prevent inappropriate immunopathology [24]. For example, the *TIR8* gene encodes for single immunoglobulin IL-1-related receptor (SIGIRR) [25]. This TLR/IL-1R family member suppresses LPS or IL-1-induced activation of NF-κB, a process that protects from immunity-mediated tissue damage upon pathogen challenge or dextran-induced damage of the intestinal epithelium [25]. Inside the kidney, SIGIRR is expressed by tubular epithelial cells, dendritic cells, and macrophages but functional activity of SIGIRR is limited to renal immune cells only [26]. SIGIRR was also shown to suppress inflammation and tissue remodeling in intrarenal immune cells during urinary tract infection [26], lupus nephritis [27], [28], and postischemic acute renal failure [29], [30].

We therefore hypothesized that SIGIRR would also suppress postobstructive tubular atrophy and renal fibrosis by inhibiting the TLR-dependent activation of renal inflammation. We addressed this hypothesis experimentally by inducing UUO in *Sigirr*(*Tir8*)-deficient mice. In addition, we performed UUO in mice deficient for either TLR2, TLR9 or MyD88.

## Results

### SIGIRR suppresses the activation of intrarenal immune cells

**Figure 1:**
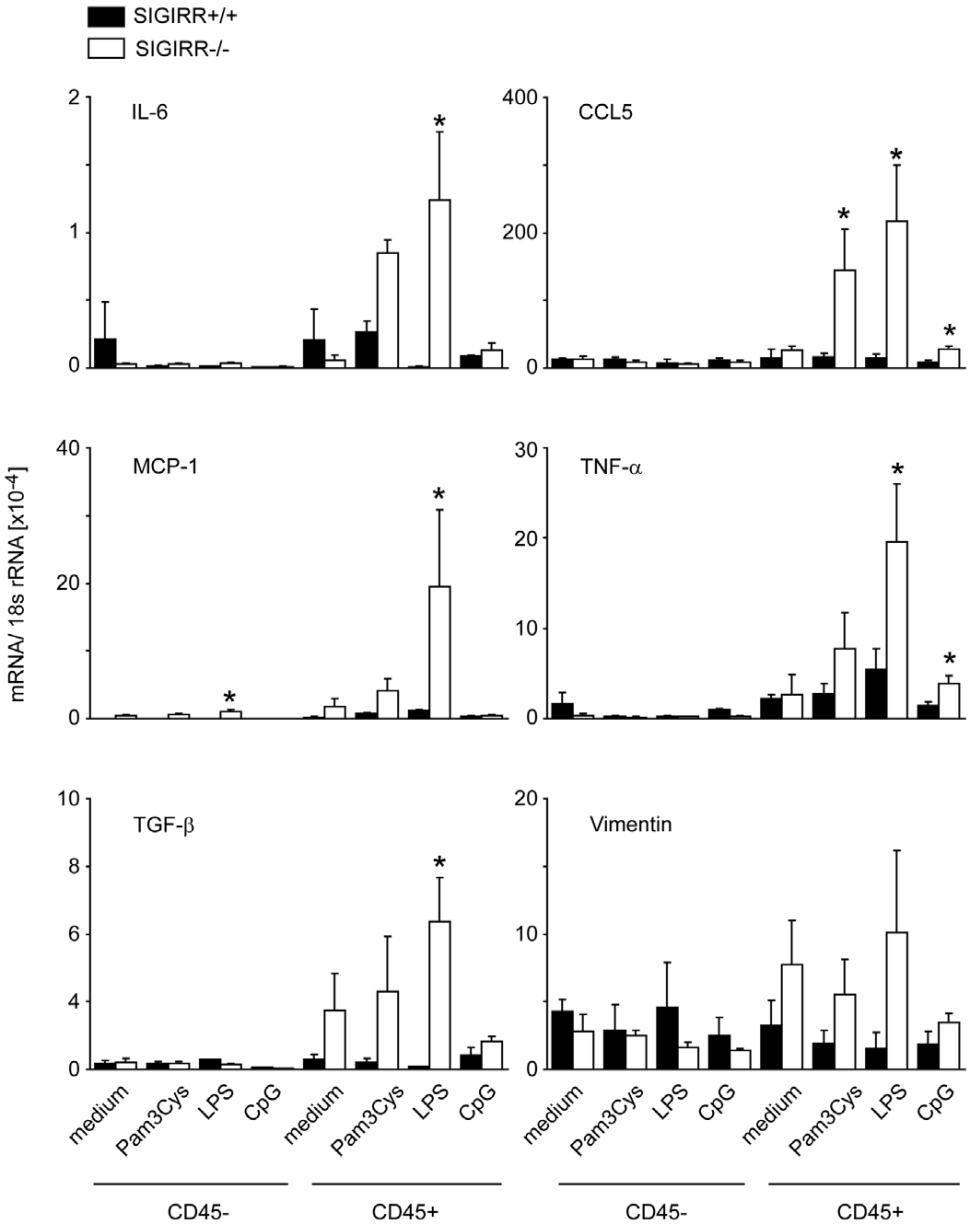
SIGIRR and the activation of renal immune and non-immune cells. Renal cell suspensions from wildtype and *Sigirr*-deficient mice were separated into CD45 positive immune cells and CD45 negative non-immune cells by magnetic bead isolation as described in methods. Cells were stimulated with 1 µg/ml Pam3Cys lipopeptide (TLR2), LPS (TLR4) or CpG-DNA (TLR9) and mRNA expression levels were determined by real-time RT-PCR for several genes after 6 hours of stimulation as indicated. Results were related to the respective 18S rRNA expression and are expressed as means ± SD. * p<0.05 versus wildtype.

SIGIRR is known to suppress TLR signaling in antigen-presenting cells [25]. Therefore, we first determined its potential to suppress LPS-induced production of proinflammatory and profibrotic mediators in renal immune and non-immune cells. We prepared single cell suspensions from kidneys of wildtype and *Sigirr*-deficient mice and separated CD45 positive immune cells and CD45 negative non-immune cells by magnetic bead isolation before all cells were incubated with either medium or medium plus LPS. Lack of SIGIRR increased the TLR agonist-induced mRNA expression of IL-6, RANTES/CCL5, MCP-1/CCL2, TNFα, and TGF-β but only in CD45 positive cells (Figure 1). Thus, SIGIRR suppresses the TLR-induced expression of proinflammatory cytokines in renal immune cells but not in renal parenchymal cells.

### Renal expression of TLR and their endogenous agonists after UUO

**Figure 2:**
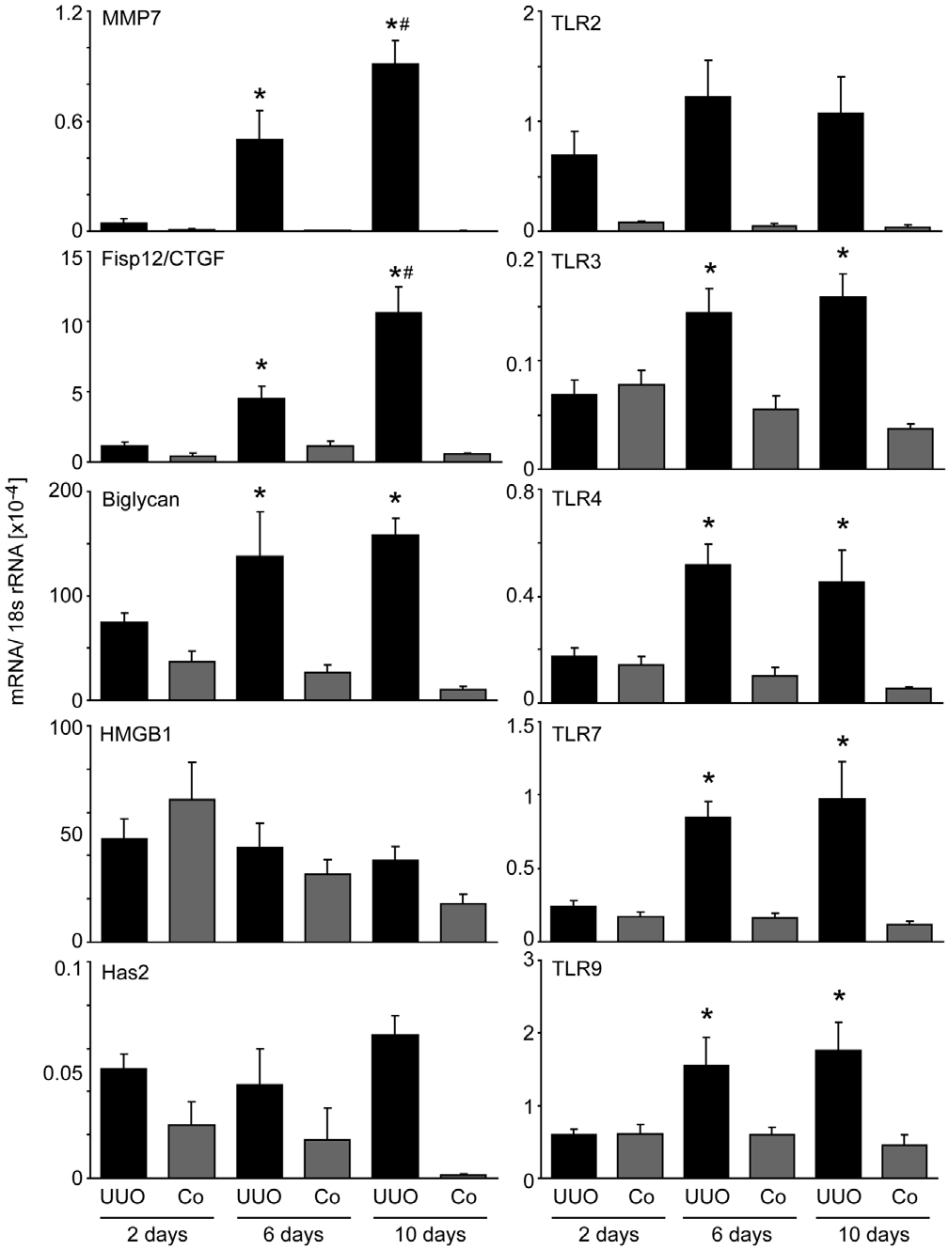
TLR and TLR agonist mRNA expression after UUO. Wildtype mice underwent surgery and UUO kidneys (black bars) or contralateral kidneys (Co, grey bars) were harvested at different time points after surgery as indicated. mRNA expression levels were determined by real-time RT-PCR and related to the respective 18S rRNA expression. Data are expressed as means ± SD. * p<0.05 versus wildtype of the same time point, # p<0.05 versus time point day 2.

Renal immune and non-immune cells express TLRs [31] but how are TLRs and their potential endogenous agonists regulated in the postobstructive kidney? To address this question we performed UUO in 6 weeks old C57BL/6 mice, harvested kidneys at 2, 6 or 10 days after surgery, and performed real-time RT-PCR to quantify mRNA expression in obstructed and sham-operated kidneys. The renal mRNA levels of fibrosis-related genes such as MMP7 or CTGF were induced in obstructed but not in sham-operated kidneys from as early as 2 days after surgery and their expression level increased over time along with the known progressive nature of interstitial fibrosis after UUO (Figure 2). A similar mRNA expression pattern was observed for biglycan, a matrix molecule that can serve as a DAMP by ligating TLR2 and TLR4 (Figure 2). By contrast, the DAMP HMGB1 was not regulated by UUO whereas the DAMP-producing enzyme hyaloronic acid synthetase (HAS)2 was induced in obstructed and sham-operated kidneys early post-surgery (Figure 2). The HAS2 induction declined over time in sham-operated but not in obstructed kidneys. Next we quantified the mRNA expression of TLR2, −3, −4, −7, and −9. TLR2 was the only receptor already induced in obstructed kidneys at day 2 after surgery (Figure 2). All TLRs were induced only in obstructed kidneys from day 6 after surgery but the expression levels did not further increase at day 10. Together, surgery increases renal DAMP mRNA expression and UUO further increases the expression levels for biglycan and HAS2. TLRs are generally induced in obstructed kidneys but the expression level dynamics do not correlate with those of genes that are known to mirror the progression of interstitial fibrosis.

### SIGIRR is expressed in the kidney during UUO

**Figure 3:**
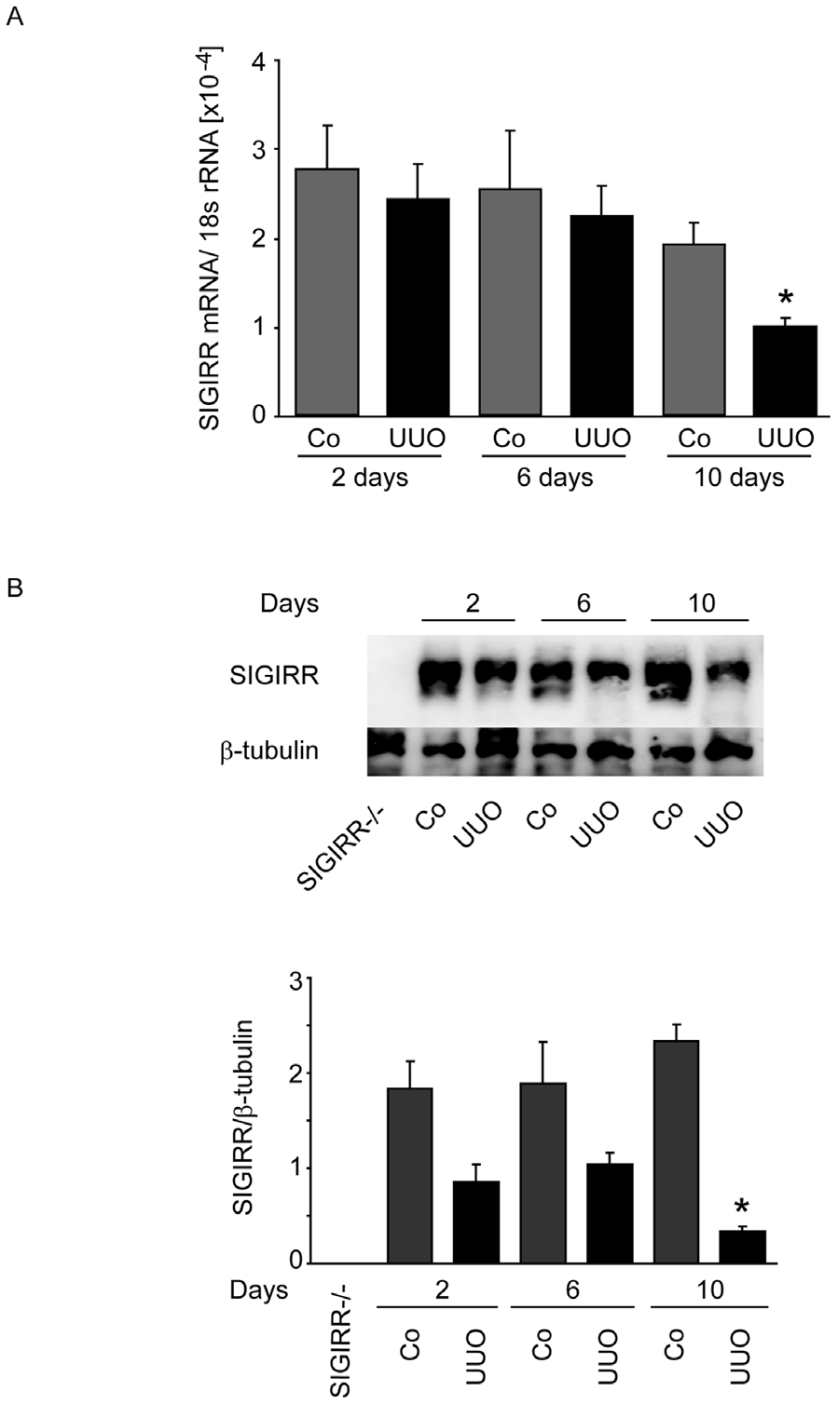
Renal SIGIRR expression after UUO. A. Wildtype mice underwent surgery and UUO kidneys (black bars) or contralateral kidneys (Co, grey bars) were harvested at different time points after surgery as indicated. mRNA expression levels were determined by real-time RT-PCR and related to the respective 18S rRNA expression. Data are expressed as means ± SD. * p<0.05 versus wildtype of the same time point, # p<0.05 versus time point day 2. B. Protein samples were obtained from the same kidney for SIGIRR Western blot. A kidney from *Sigirr*-deficient mouse served as negative control for the specificity of the SIGIRR antibody. β-tubulin staining is given as a loading control. The quantitative analysis is shown below. *p<0.05 versus contralateral kidney.

As UUO induced the intrarenal expression of many genes related to TLR signaling we questioned whether UUO would also induce renal SIGIRR expression. However, renal mRNA expression of SIGIRR rather declined in UUO versus unobstructed kidneys, especially 10 days after UUO (Figure 3A). Western blot confirmed this downmodulation of renal SIGIRR expression in UUO kidneys 10 days after surgery (Figure 3B). Thus, the intrarenal induction of TLR expression after UUO is associated with a downregulation of SIGIRR.

### Lack of SIGIRR does not affect the renal expression of proinflammatory and profibrotic mediators during UUO

**Figure 4:**
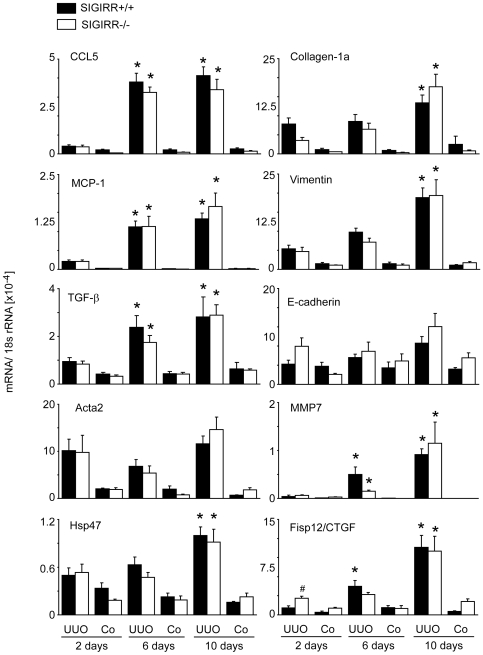
Renal mRNA expression in wildtype and *Sigirr*-deficient kidneys after after UUO. Wildtype (black bars) and *Sigirr*-deficient mice (white bars) underwent UUO and kidneys were harvested at different time points after surgery as indicated. mRNA expression levels were determined by real-time RT-PCR and related to the respective 18S rRNA expression. Data are expressed as means ± SD. # p<0.05 versus wildtype of the same time point, * p<0.05 versus time point day 2.

As SIGIRR suppresses the production of proinflammatory mediators in intrarenal immune cells upon exposure to TLR agonists, we expected higher cytokine expression levels in UUO kidneys of *Sigirr*-deficient mice. But when we compared renal mRNA expression levels of MCP-1/CCL2 and RANTES/CCL5 in obstructed and sham-operated kidneys at 2, 6 or 10 days after surgery we found their expression levels to be independent of the SIGIRR genotype (Figure 4). The same was found for the profibrotic cytokines TGF-β and CTGF or for collagen-1α, vimentin, Acta2/SMA (Figure 4). The mRNA expression of E-cadherin, Hsp47 and MMP7 was also independent of the genotype. Together, lack of SIGIRR does not affect the intrarenal mRNA expression of the proinflammatory chemokines, profibrotic mediators, or markers of renal fibrosis after UUO.

### Lack of SIGIRR does not affect macrophage recruitment or myofibroblasts in UUO

**Figure 5:**
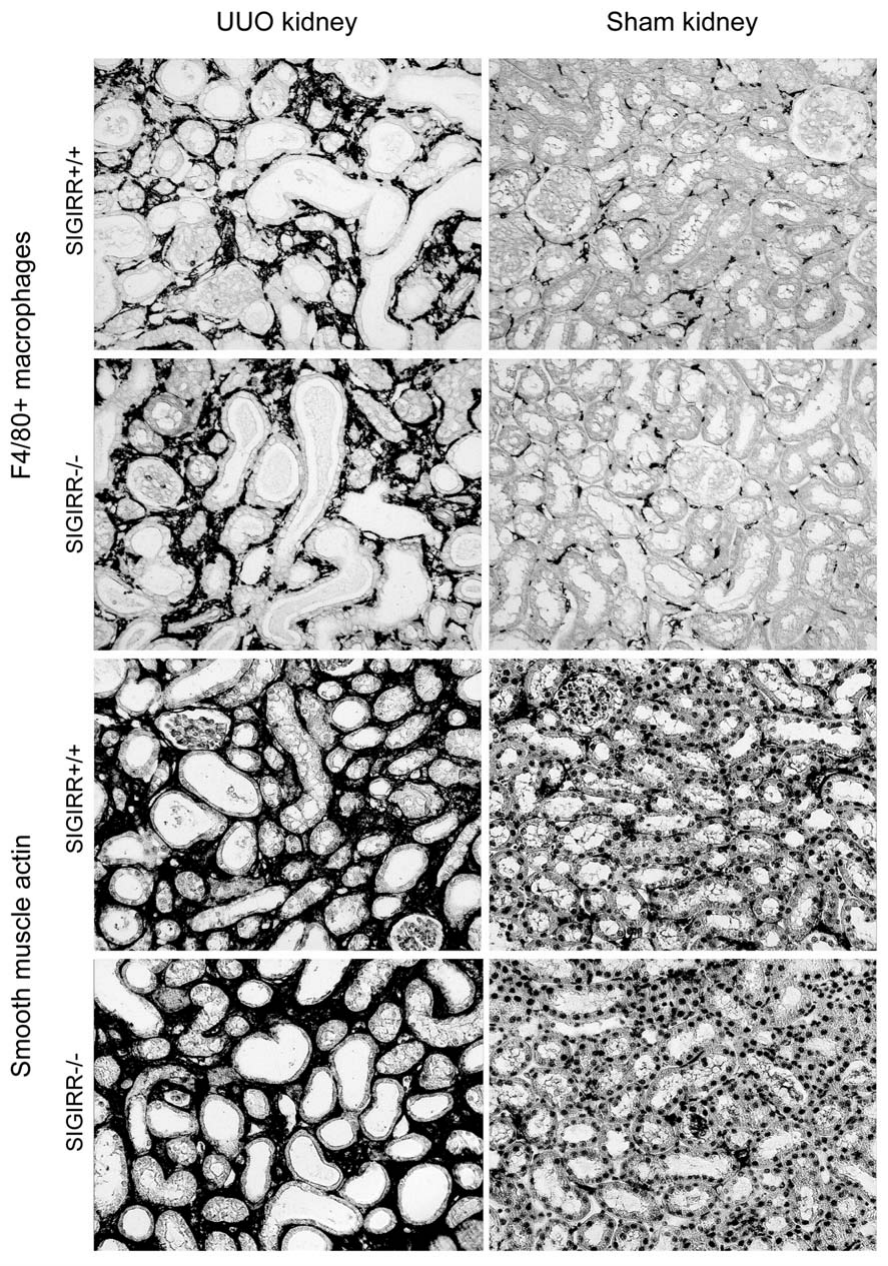
Macrophages and myofibroblasts in the postobstructive kidney. Cortical renal sections were either stained with F4/80 or SMA. Images illustrate representative sections of UUO and sham-operated kidneys 10 days after UUO in mice of the respective group as indicated (original magnification 200×).

The intrarenal expression of proinflammatory chemokines should correspond to the number of infiltrating leukocytes into the postobstructive kidney. Immunostaining for F4/80 positive macrophages revealed no significant difference in the F4/80 positive areas between UUO (or sham) kidneys from *Sigirr*-deficient and wildtype mice (8.0±0.7% versus 7.6±0.7%, Figure 5). Intrarenal macrophages and the expression of profibrotic mediators usually predict the number of myofibroblast after UUO. We quantified intrarenal myofibroblasts by immunostaining for smooth muscle actin (SMA) but could again not detect any difference in the SMA positive areas between UUO (or sham) kidneys from *Sigirr*-deficient and wildtype mice (21.7±2.1% versus 22.0±1.8%, Figure 5). Thus, lack of SIGIRR does not affect the numbers of intrarenal macrophages and myofibroblasts in postobstructive kidneys.

### Lack of SIGIRR does not affect tubular atrophy and interstitial fibrosis during UUO

**Figure 6:**
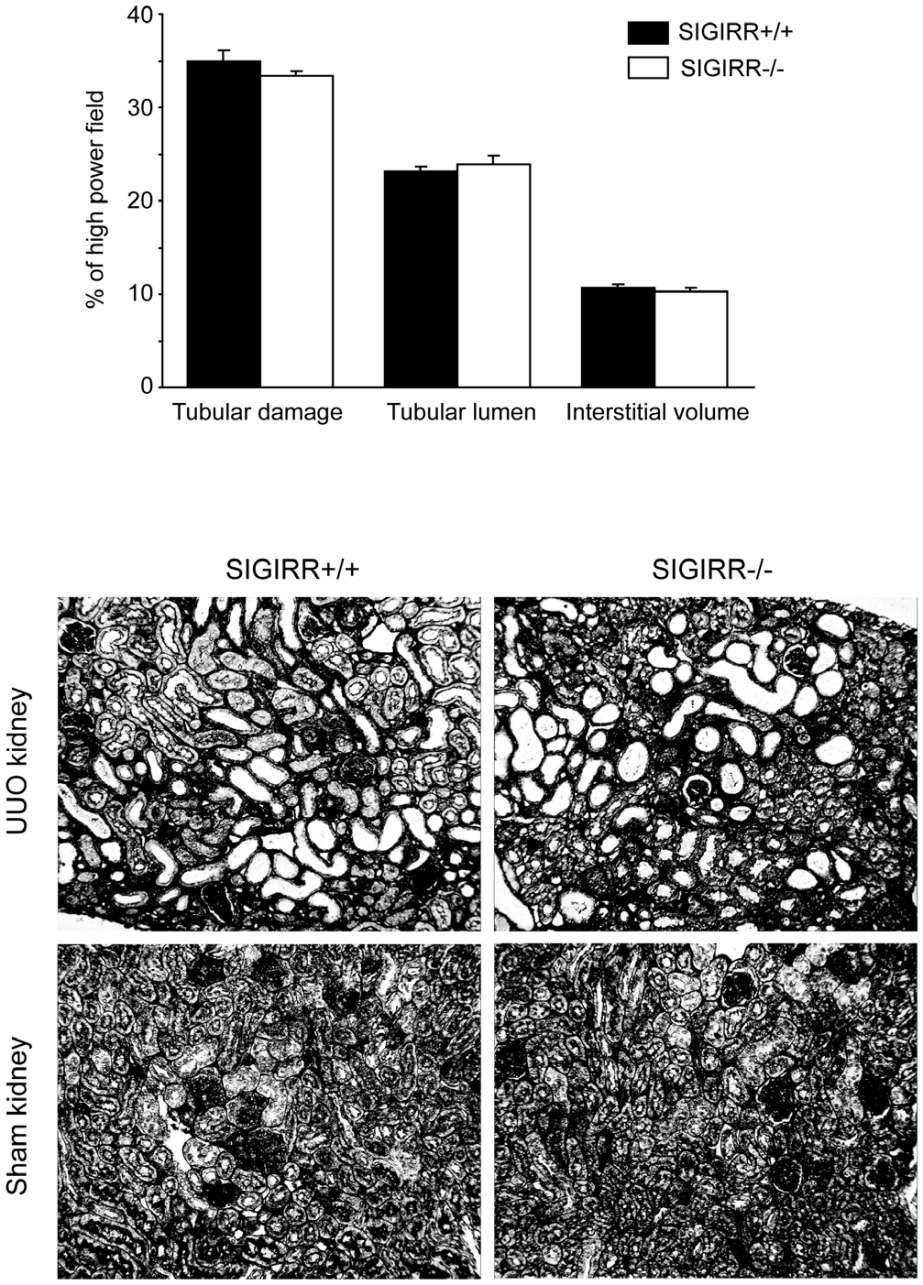
SIGIRR and morphometric analysis of tubulointerstitial pathology after UUO. Cortical renal sections were stained with silver. Images illustrate representative sections of UUO and sham-operated kidneys 10 days after UUO in mice of the respective group as indicated (original magnification 200×). The indices for interstitial volume, tubular damage, and tubular lumen were determined in UUO kidneys from wildtype (black bars) and *Sigirr*-deficient mice (white bars) by quantitative morphometry as decribed in the methods section. Values represent means ± SD from 10 high power fields of UUO kidneys from 10 mice in each group.

Intrarenal macrophages and myofibroblasts contribute to progressive tissue remodeling in the postobstructive kidney. We used morphometry of silver-stained renal sections to quantify tubular atrophy, tubular dilatation, and interstitial volume as markers of obstructive tubulointerstitial disease in both strains of mice. None of these parameters was differed significantly between UUO (or sham) kidneys from *Sigirr*-deficient and wildtype mice (Figure 6). Thus, lack of SIGIRR does neither affect intrarenal inflammation, macrophage or myofibroblast accumulation or tissue remodeling after UUO.

### Postobstructive kidney remodeling develops independent of MyD88, Tlr2, and Tlr9

**Figure 7:**
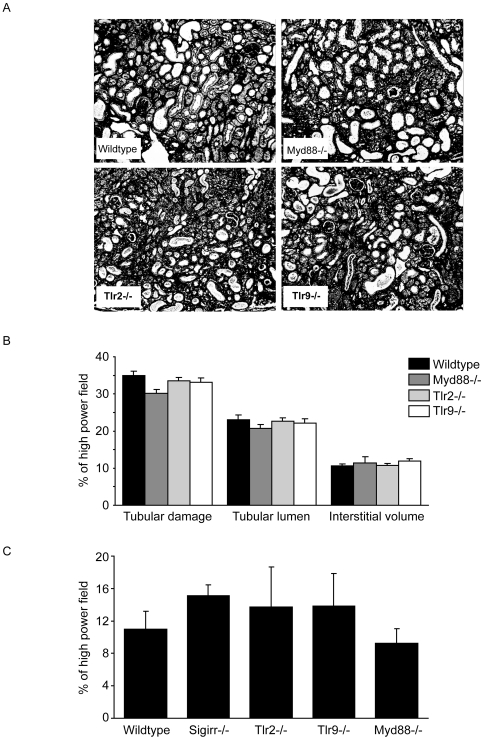
TLRs and morphometric analysis of tissue remodeling after UUO. A: Cortical renal sections were stained with silver. Images illustrate representative sections of UUO kidneys 10 days after UUO in mice of the respective group as indicated (original magnification 200×). B: The indices for tubular cell damage, tubular dilatation, interstitial volume were determined by quantitative morphometry in obstructed kidneys at 10 days as described in methods. C: Interstitial fibrosis was quantified by digital morphometry on Masson Trichrom-stained sections as percentage of entire high power field. Values represent means ± SD from 10–15 high power fields.

SIGIRR is known to suppress innate immunity via interaction with the intracellular adaptor molecule MyD88 which blocks the activation of multiple TLRs and IL-1Rs [32]. The lacking phenotype of *Sigirr*-deficient mice suggests that innate immune activation via MyD88 does not contribute or is redundant in the UUO model. To test this hypothesis we also induced UUO in mice deficient for MyD88, TLR2, and TLR9, and evaluated tubulointerstitial remodeling at 10 days after UUO compared to a separate set of wildtype controls. Morphometry of silver and Masson Trichrome stains did not reveal any significant differences for the indices of tubular damage, tubular lumen, interstitial volume (Silver), and interstitial fibrosis (Masson Trichrome) of any of the aforementioned mouse strains as compared to wildtype mice (Figure 7). Thus, neither SIGIRR, MyD88, TLR2 nor TLR9 significantly contribute to the remodeling of the tubulointerstitium 10 days after UUO.

### TLR activation does not trigger TGF-β, SMA and collagen-1a production in renal fibroblasts

**Figure 8:**
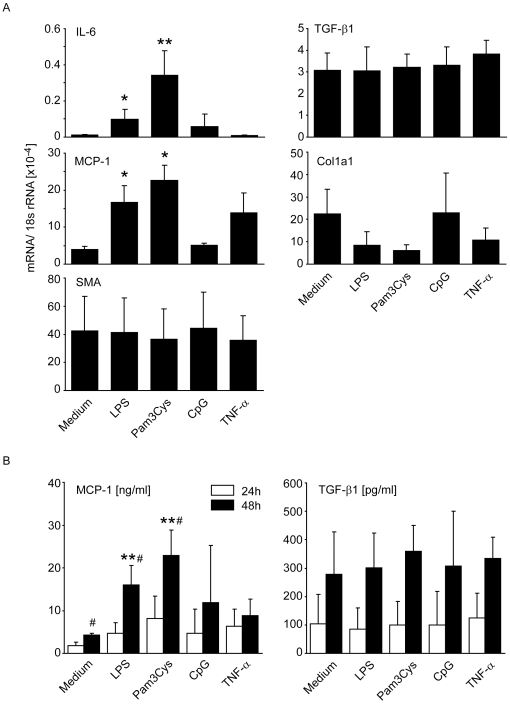
TLR-induced expression of cytokines and profibrotic mediators in renal fibroblasts. CD90+ fibroblasts were prepared from mice and cultured as described in methods. The cells were stimulated with 1 ug/ml of either Pam3Cys lipopeptide (TLR2 agonist), LPS (TLR4 agonist), CpG-DNA (TLR9 agonist) or 5 ng/ml TNFα. A: After 24 hours of stimulation cell mRNA was isolated and quantified by real-time PCR and refered to the respective 18S rRNA level. Data represent means ± SEM from three independent experiments. * p<0.05 versus medium. B: Renal fibroblasts were stimulated as before and cell culture supernatants were obtained after 24 (white bars) and 48 hours of stimulation (black bars). MCP-1 and TGF-β levels were measured by ELISA. Data represent means ± SEM from three independent experiments. * p<0.05 versus medium.

The capacity of TLRs to activate renal immune and non-immune cells for the secretion of proinflammatory cytokines is well described [31]. It is, however, yet unknown whether TLR signaling modulates the secretion of profibrotic mediators. To address this issue we isolated CD90+ renal fibroblasts from 6 week old mice and stimulated them with agonists for TLR2 (Pam3Cys lipopetide), TLR4 (LPS), TLR9 (CpG-DNA) and with TNF-α. The activation of TLR2 and TLR4 activated renal fibroblasts to induce MCP-1/CCL2 and IL-6 mRNA within 24 hours while TGF-β, collagen-1α, and smooth muscle actin remained at baseline levels (Figure 8A). The CpG-DNA had no significant effect on MCP-1 mRNA expression because renal fibroblasts do not express TLR9. The same pattern was found at the protein level by MCP-1/CCL2 and TGF-β ELISA when we analysed renal fibroblast culture supernatants after 48 hours of stimulations (Figure 8B). Thus, TLR activation in renal fibroblasts triggers proinflammatory mediators like MCP-1/CCL2 and IL-6 but does not induce the production of profibrotic molecules like TGF-ß, collagen-1α, and smooth muscle actin in renal fibroblasts.

## Discussion

We had hypothesized that SIGIRR would suppress postobstructive tubular atrophy and renal fibrosis by inhibiting the TLR-dependent activation of renal inflammation and fibrosis. Our results are in contrast to this hypothesis and rather document that UUO-induced kidney remodeling is independent of the *Sigirr* genotype as well as of TLR2, TLR9, and MyD88 compared to wildtype C57BL/6 mice.

Lack of SIGIRR was recently shown to aggravate a number of different kidney disease models including lupus nephritis [27], [28], renal infection [26], and postischemic acute renal failure [29], [30]. In lupus nephritis lack of SIGIRR mainly affected the systemic autoimmune disease but in renal infection or postischemic renal failure intrarenal dendritic cells or macrophages were shown to be increasingly activated in *Sigirr*-deficient mice [26], [29], [30]. Increased activation of antigen-presenting cells increased intrarenal cytokine signaling, leukocyte recruitment, and renal injury [26], [30]. The suppressive role of SIGIRR on immunopathology in these models could be refered to the role of TLR signaling during immune recognition of LPS from uropathogenic *E. coli* [26] or DAMP release during ischemic cell necrosis [30], two well known triggers of TLR signaling [8], [10]. In fact, the role of TLR4 signaling in intrarenal dendritic cells was documented to contribute to the immunopathology infective pyelonephritis in mice [33]. Lack of SIGIRR's inhibitory effect of TLR4 signaling aggravates inflammation and immunopathology in renal infection [26]. Several studies document a similar role for TLR2, TLR4, and MyD88 in postischemic acute renal failure of C57BL/6 mice [12], [13], [14]. Hence, lack of SIGIRR's inhibitory effect on this signaling pathway aggravates postischemic acute renal failure [30].

Our finding that lack of SIGIRR does not modulate UUO-induced renal pathology question a functional contribution of TLR signaling in UUO. Leemans, *et al.* reported results from UUO experiments in *Tlr2*-deficient mice of the C57BL/6 background [22]. Tubular injury and interstitial fibrosis remained unaffected by the *Tlr2* genotype which is consistent with our findings as well as with our results obtained with *Myd88*-deficient mice. The latter data are confirmative because MyD88 is the only signaling adaptor for TLR2 [31]. MyD88 is also the only signaling adaptor for the CpG-DNA receptor TLR9, hence, *Tlr9*-deficient mice also had no phenotype in the UUO model. All these data are consistent with the lack of phenotype in *Sigirr*-deficient mice because SIGIRR suppresses TLR signaling by inhibiting TIR domains interactions between the TLRs and MyD88 [32]. We therefore conclude that SIGIRR is redundant in UUO-induced renal fibrosis because TLR2-, TLR9- and MyD88 signaling do not significantly contribute to this model. Two other studies have documented that *Tlr4*-deficient mice develop less renal fibrosis after UUO [23], [34]. These data are not necessarily conflicting to our results because MyD88 is not the only signaling adaptor for TLR4 [31]. TLR4 does also induce inflammation via its adaptor TRIF and the role of SIGIRR on TRIF signaling is not yet fully characterized [25]. However, it is of note that we (data not shown) and others [35] were unable to reproduce the strong UUO phenotype of Tlr4-deficient mice reported by Campbell, *et al.* [23] or not even their weak UUO phenotype reported by Pulskens, *et al.* [34]. Impaired TRIF rather than MyD88 signaling might account for less renal fibrosis in *Tlr4*-deficient mice [23]. In fact, TRIF signaling was found to be of negligible importance in nephrotoxic serum nephritis [36] but contributed to chronic allograft nephropathy in mice [19].

In summary, tubular atrophy and interstitial fibrosis after UUO develop independent of the TLR signaling inhibitor SIGIRR mainly because TLR2, TLR9, and their essential adaptor molecule and SIGIRR's interaction target MyD88 do not significantly contribute to the postobstructive renal inflammation and tissue remodeling. Obviously, TLR-MyD88 signaling, known to mediate acute kidney injuries, is not significantly involved in postobstructive progressive renal fibrosis.

## Materials and Methods

### Animal studies

*Sigirr*-deficient mice were generated and backcrossed into the C57BL/6 background for 10 generations as described [37]. Mice deficient for MyD88, TLR2, and TLR9 were generated by Prof. Dr. Shizuo Akira, Osaka, Japan as described [38], [39], [40] and backcrossed to C57BL/6 for at least 6 generations. We used C57BL/6 mice from Charles River (Sulzfeld, Germany) for backcrossing and as wildtype controls. All mice were housed in groups of 4 mice in filter top cages with a 12 hour dark/light cycle and unlimited access to food and water. Cages, bedding, nestlets, food and water were sterilized by autoclaving before use. UUO was performed in 6–8 week old, sex- and age-matched wildtype and gene-deficient mice as previously described [41]. Contralateral kidneys served as intraindividual control. All experimental procedures were performed according to the German animal care and ethics legislation and had been approved by the local government authorities (Regierung von Oberbayern, Az 55.2-1-54-2531-34-03 and 11-10).

### Morphological evaluation

Tissue sections from the obstructed and the contralateral kidneys of each mouse were fixed in 10% formalin in PBS and embedded in paraffin. 2 µm sections were stained with periodic acid-Schiff reagent and silver as standard procedures. To count interstitial cells 15 high power fields (hpf, 400×) were analyzed by a blinded observer. Positive cells were counted per high power field omitting positive cells in glomerular fields. Morphometry of tubulointerstitial was performed as previously described [42]. In brief, the interstitial volume index was determined by superposing a grid containing 100 (10×10) sampling points on photographs of 10 non-overlapping cortical fields of silver stained tissue (400×) of each kidney. The number of points overlying interstitial space were counted. The indices of tubular cell damage and tubular dilatation were assessed accordingly. Interstitial fibrosis was quantified by digital morphometry on Masson Trichrome-stained paraffin-embedded sections as described [43]. The following primary antibodies were used: rat anti-F4/80 (Serotec, Oxford, UK, 1∶50), mouse-anti-smooth muscle actin (Dako, Glostrup, Denmark, 1∶100).

### *In vitro* studies

For CD45+ and CD45- renal cells isolation kidneys were minced, passed through 70 and 30 µm cell strainers, and positive selection of CD45+ cells was performed by magnetic cell sorting technique (MACS separation, Miltenyi, Bergisch-Gladbach, Germany) as previously described [44]. Cells were incubated with the magnetic beads in 500 µl buffer containing phosphate-buffered saline (PBS) pH 7.2, 0.5% BSA and 2 mM EDTA at 4°C for 15 min. The cells were washed by adding 1–2 ml of buffer and centrifuged twice. Pellets containing labeled cells were resuspended in buffer and passed through MACS LS column. After separation cells were found to be >95% purity by FACS analysis. The negative selected cells (flow through) were used as CD45- cell fraction. The CD45+ cells were cultured in RPMI medium whereas the CD45- cells were cultured in Dublecco's modified Eagle medium (DMEM). Both media were supplemented with 10% fetal calf serum (FCS) and 1% penicillin-streptomycin 100 U/ml and 100 µg/ml. The renal CD45+ or CD45- cells were treated with either medium or ultrapure LPS, Pam3Cys lipopeptide, or CpG-DNA (all Invivogen, San Diego, CA). Total RNA and cell culture supernatants were harvested after 24 or hours for real time RT-PCR. For fibroblasts isolation kidneys were minced, passed through 70 and 30 µm cell strainers, and incubated in normal cell culture medium at 37°C, 5% CO_2_ for one to two hours to deplete monocytes and lymphocytes. The cells were cultured in DMEM supplemented with 10% FCS and 1% penicillin-streptomycin 100 U/ml and 100 µg/ml. After several passages until overgrowing of fibroblasts, positive selection of fibroblasts was isolated by magnetic cell sorting technique (MACS separation, Miltenyi, Bergisch-Gladbach, Germany) as previously described [44]. Cells were stained with primary PE-conjugated antibody (monoclonal anti-mouse CD90, Clone 30-H12, Acris Antibodies, Hiddenhausen, Germany) in 100 µl buffer containing PBS, pH 7.2, 0.5% BSA and 2 mM EDTA at 4°C for 10 min in the dark. The cells were washed by adding 1–2 ml of buffer and centrifuged twice. Pellets were resuspended in buffer mixed with anti-PE microbeads and incubated at 4°C for 15 min in the dark. The collection of labeled cells was carried out by passing through MACS LS column. After separation cells were found to be >95% purity by FACS analysis. The renal fibroblasts were treated with either medium or various stimuli; Pam3CysSK4, ultrapure LPS (both Invivogen), CpG-ODN 1668 (TIB Molbiol, Berlin, Germany), and TNF-α (ImmunTools, Firesoythe, Germany. Total RNA and cell culture supernatants were harvested after 24 or 48 hours for real-time RT-PCR and ELISA, respectively.

### RNA preparation and real-time quantitative (TaqMan) RT-PCR

Reverse transcription and real-time RT-PCR from total renal or cell RNA was performed as described [28]. SYBR Green Dye detection system was used for quantitative real-time PCR on a Light Cycler 480 (Roche, Mannheim, Germany). Gene-specific primers (300 nM, Metabion, Martinsried, Germany) were used as listed in table 1. Controls consisting of ddH2O were negative for target and housekeeper genes.

**Table 1:** Primers used for real-time RT-PCR.

Gene	Accession no.	Sequence
*Sigirr*	NM023059	forward primer: 5′- AGAGGTCCCAGAAGAGCCAT -3′reverse primer: 5′- AAGCAACTTCTCTGCCAAGG - 3′
*Ccl2/Mcp-1*	NM011333	forward primer: 5′- CCTGCTGTTCACAGTTGCC -3′reverse primer: 5′- ATTGGGATCATCTTGCTGGT-3′
*Acta2/α-Sma*	NM_007392	forward primer: 5′- ACTGGGACGACATGGAAAAG -3′reverse primer: 5′- GTTCAGTGGTGCCTCTGTCA - 3′
*Col1a1*	NM_007742	forward primer: 5′- ACATGTTCAGCTTTGTGGACC -3′reverse primer: 5′- TAGGCCATTGTGTATGCAGC - 3′
*Tgf-β1*	NM_011577	forward primer: 5′- GGAGAGCCCTGGATACCAAC -3′reverse primer: 5′- CAACCCAGGTCCTTCCTAAA -3′
*Il-6*	NM031168	forward primer: 5′- TGATGCACTTGCAGAAAACA -3′reverse primer: 5′- ACCAGAGGAAATTTTCAATAGGC - 3′
*Tnf-α*	NM011609	forward primer: 5′- CCACCACGCTCTTCTGTCTAC -3′reverse primer: 5′- AGGGTCTGGGCCATAGAACT -3′
*Ccl5/Rantes*	NM_013653	forward primer: 5′- CCACTTCTTCTCTGGGTTGG-3′reverse primer: 5′- GTGCCCACGTCAAGGAGTAT-3′
*Vimentin*	NM_011701	forward primer: 5′- AGAGAGAGGAAGCCGAAAGC-3′reverse primer: 5′- TCCACTTTCCGTTCAAGGTC-3′
*E-cadherin*	NM_009864	forward primer: 5′- GAGGTCTACACCTTCCCGGT-3′reverse primer: 5′- CCACTTTGAATCGGGAGTCT-3′
*Mmp7*	NM_010810	forward primer: 5′- CACATCAGTGGGAACAGGC-3′reverse primer: 5′- AGTTTTCCAGTCATGGGCAG-3′
*Hsp47*	NM_009825	forward primer: 5′- AACTGTCACTTGCCTGGGTT-3′reverse primer: 5′- TAAGGTGCCCAGAAGGAGAG-3′
*Fsp12/Ctgf*	NM_010217	forward primer: 5′- AGCTGACCTGGAGGAAAACA-3′reverse primer: 5′- CCGCAGAACTTAGCCCTGTA-3′
*Biglycan*	NM_007542	forward primer: 5′- CTTGCTGCCCTTATCCTCTG-3′reverse primer: 5′- GGAAGCATAACAGGGTTGGA-3′
*Hmgb1*	NM_010439	forward primer: 5′- TGAGCTCCATAGAGACAGCG-3′reverse primer: 5′- AGGATCTCCTTTGCCCATGT-3′
*Has2*	NM_008216	forward primer: 5′- ATAAGCGGTCCTCTGGGAAT-3′reverse primer: 5′- GTTGGTAAGGTGCCTGTCGT-3′
*Tlr2*	NM_011905	forward primer: 5′- CATCACCGGTCAGAAAACAA-3′reverse primer: 5′- ACCAAGATCCAGAAGAGCCA-3′
*Tlr3*	NM_126166	forward primer: 5′- ATGATACAGGGATTGCACCC-3′reverse primer: 5′- ATAGGGACAAAAGTCCCCCA-3′
*Tlr4*	NM_021297	forward primer: 5′- TGTTCTTCTCCTGCCTGACA-3′reverse primer: 5′- TGTCATCAGGGACTTTGCTG-3′
*Tlr7*	NM_133211	forward primer: 5′- GGATGATCCTGGCCTATCTC-3′reverse primer: 5′- TGTCTCTTCCGTGTCCACAT-3′
*Tlr9*	NM_031178	forward primer: 5′- CAGTTTGTCAGAGGGAGCCT-3′reverse primer: 5′- CTGTACCAGGAGGGACAAGG-3′
*18s RNA*	NR003278	forward primer: 5′- GCAATTATTCCCCATGAACG-3′reverse primer: 5′- AGGGCCTCACTAAACCATCC- 3′

### Statistical analysis

Data were expressed as mean ± standard error of the mean (SEM). Data from wild-type and *Sigirr*-deficient mice were compared with ANOVA on ranks followed by the Student-Newman-Keuls test using SigmaStat Software (Jandel Scientific, Erkrath, Germany). *T*-test was used for direct comparisons between wild-type and gene-deficient mice. Data are expressed as mean values ± SEM. A p value less than 0.05 indicated statistical significance.

## References

[1] Zeisberg M, Neilson EG (2010). Mechanisms of tubulointerstitial fibrosis.. J Am Soc Nephrol.

[2] Nangaku M (2006). Chronic hypoxia and tubulointerstitial injury: a final common pathway to end-stage renal failure.. J Am Soc Nephrol.

[3] Liu Y (2010). New insights into epithelial-mesenchymal transition in kidney fibrosis.. J Am Soc Nephrol.

[4] Wada T, Sakai N, Matsushima K, Kaneko S (2007). Fibrocytes: a new insight into kidney fibrosis.. Kidney Int.

[5] Rosenbloom J, Castro SV, Jimenez SA (2010). Narrative review: fibrotic diseases: cellular and molecular mechanisms and novel therapies.. Ann Intern Med.

[6] Ninichuk V, Anders HJ (2008). Bone marrow-derived progenitor cells and renal fibrosis.. Front Biosci.

[7] Medzhitov R (2008). Origin and physiological roles of inflammation.. Nature.

[8] Akira S (2009). Innate immunity to pathogens: diversity in receptors for microbial recognition.. Immunol Rev.

[9] Anders HJ (2010). Toll-like receptors and danger signaling in kidney injury.. J Am Soc Nephrol.

[10] Kono H, Rock KL (2008). How dying cells alert the immune system to danger.. Nat Rev Immunol.

[11] Anders HJ, Schlondorff D (2007). Toll-like receptors: emerging concepts in kidney disease.. Curr Opin Nephrol Hypertens.

[12] Leemans JC, Stokman G, Claessen N, Rouschop KM, Teske GJ (2005). Renal-associated TLR2 mediates ischemia/reperfusion injury in the kidney.. J Clin Invest.

[13] Wu H, Chen G, Wyburn KR, Yin J, Bertolino P (2007). TLR4 activation mediates kidney ischemia/reperfusion injury.. J Clin Invest.

[14] Shigeoka AA, Holscher TD, King AJ, Hall FW, Kiosses WB (2007). TLR2 is constitutively expressed within the kidney and participates in ischemic renal injury through both MyD88-dependent and -independent pathways.. J Immunol.

[15] Rusai K, Sollinger D, Baumann M, Wagner B, Strobl M (2010). Toll-like receptors 2 and 4 in renal ischemia/reperfusion injury.. Pediatr Nephrol.

[16] Cunningham PN, Wang Y, Guo R, He G, Quigg RJ (2004). Role of Toll-like receptor 4 in endotoxin-induced acute renal failure.. J Immunol.

[17] Zhang B, Ramesh G, Uematsu S, Akira S, Reeves WB (2008). TLR4 signaling mediates inflammation and tissue injury in nephrotoxicity.. J Am Soc Nephrol.

[18] Yasuda H, Leelahavanichkul A, Tsunoda S, Dear JW, Takahashi Y (2008). Chloroquine and inhibition of Toll-like receptor 9 protect from sepsis-induced acute kidney injury.. Am J Physiol Renal Physiol.

[19] Wang S, Schmaderer C, Kiss E, Schmidt C, Bonrouhi M (2010). Recipient Toll-like receptors contribute to chronic graft dysfunction by both MyD88- and TRIF-dependent signaling.. Dis Model Mech.

[20] Bascands JL, Schanstra JP (2005). Obstructive nephropathy: insights from genetically engineered animals.. Kidney Int.

[21] Chevalier RL, Forbes MS, Thornhill BA (2009). Ureteral obstruction as a model of renal interstitial fibrosis and obstructive nephropathy.. Kidney Int.

[22] Leemans JC, Butter LM, Pulskens WP, Teske GJ, Claessen N (2009). The role of Toll-like receptor 2 in inflammation and fibrosis during progressive renal injury.. PLoS One.

[23] Campbell MT, Hile KL, Zhang H, Asanuma H, Vanderbrink BA (2009). Toll-Like Receptor 4: A Novel Signaling Pathway During Renal Fibrogenesis.. J Surg Res.

[24] O'Neill LA (2008). When signaling pathways collide: positive and negative regulation of toll-like receptor signal transduction.. Immunity.

[25] Garlanda C, Anders HJ, Mantovani A (2009). TIR8/SIGIRR: an IL-1R/TLR family member with regulatory functions in inflammation and T cell polarization.. Trends Immunol.

[26] Lech M, Garlanda C, Mantovani A, Kirschning CJ, Schlondorff D (2007). Different roles of TiR8/Sigirr on toll-like receptor signaling in intrarenal antigen-presenting cells and tubular epithelial cells.. Kidney Int.

[27] Lech M, Kulkarni OP, Pfeiffer S, Savarese E, Krug A (2008). Tir8/Sigirr prevents murine lupus by suppressing the immunostimulatory effects of lupus autoantigens.. J Exp Med.

[28] Lech M, Skuginna V, Kulkarni OP, Gong J, Wei T (2009). Lack of SIGIRR/TIR8 aggravates hydrocarbon oil-induced lupus nephritis.. J Pathol.

[29] Noris M, Cassis P, Azzollini N, Cavinato R, Cugini D (2009). The Toll-IL-1R member Tir8/SIGIRR negatively regulates adaptive immunity against kidney grafts.. J Immunol.

[30] Lech M, Avila-Ferrufino A, Allam R, Segerer S, Khandoga A (2009). Resident dendritic cells prevent postischemic acute renal failure by help of single Ig IL-1 receptor-related protein.. J Immunol.

[31] Anders HJ (2007). Innate pathogen recognition in the kidney: toll-like receptors, NOD-like receptors, and RIG-like helicases.. Kidney Int.

[32] Gong J, Wei T, Stark RW, Jamitzky F, Heckl WM (2009). Inhibition of Toll-like receptors TLR4 and 7 signaling pathways by SIGIRR: A computational approach.. J Struct Biol.

[33] Patole PS, Schubert S, Hildinger K, Khandoga S, Khandoga A (2005). Toll-like receptor-4: renal cells and bone marrow cells signal for neutrophil recruitment during pyelonephritis.. Kidney Int.

[34] Pulskens WP, Rampanelli E, Teske GJ, Butter LM, Claessen N (2010). TLR4 promotes fibrosis but attenuates tubular damage in progressive renal injury.. J Am Soc Nephrol.

[35] Chowdhury P, Sacks SH, Sheerin NS (2010). Endogenous ligands for TLR2 and TLR4 are not involved in renal injury following ureteric obstruction.. Nephron Exp Nephrol.

[36] Lichtnekert J, Vielhauer V, Zecher D, Kulkarni OP, Clauss S (2009). Trif is not required for immune complex glomerulonephritis: dying cells activate mesangial cells via Tlr2/Myd88 rather than Tlr3/Trif.. Am J Physiol Renal Physiol.

[37] Garlanda C, Riva F, Polentarutti N, Buracchi C, Sironi M (2004). Intestinal inflammation in mice deficient in Tir8, an inhibitory member of the IL-1 receptor family.. Proc Natl Acad Sci U S A.

[38] Kawai T, Adachi O, Ogawa T, Takeda K, Akira S (1999). Unresponsiveness of MyD88-deficient mice to endotoxin.. Immunity.

[39] Takeuchi O, Hoshino K, Kawai T, Sanjo H, Takada H (1999). Differential roles of TLR2 and TLR4 in recognition of gram-negative and gram-positive bacterial cell wall components.. Immunity.

[40] Hemmi H, Takeuchi O, Kawai T, Kaisho T, Sato S (2000). A Toll-like receptor recognizes bacterial DNA.. Nature.

[41] Higgins DF, Lappin DW, Kieran NE, Anders HJ, Watson RW (2003). DNA oligonucleotide microarray technology identifies fisp-12 among other potential fibrogenic genes following murine unilateral ureteral obstruction (UUO): modulation during epithelial-mesenchymal transition.. Kidney Int.

[42] Ninichuk V, Khandoga AG, Segerer S, Loetscher P, Schlapbach A (2007). The role of interstitial macrophages in nephropathy of type 2 diabetic db/db mice.. Am J Pathol.

[43] Ninichuk V, Gross O, Reichel C, Khandoga A, Pawar RD (2005). Delayed chemokine receptor 1 blockade prolongs survival in collagen 4A3-deficient mice with Alport disease.. J Am Soc Nephrol.

[44] Clayton A, Steadman R, Williams JD (1997). Cells isolated from the human cortical interstitium resemble myofibroblasts and bind neutrophils in an ICAM-1–dependent manner.. J Am Soc Nephrol.

